# Successful Treatment of Acute Limb Ischemia Secondary to Iatrogenic Distal Embolization Using Catheter Directed Aspiration Thrombectomy

**DOI:** 10.3389/fsurg.2020.00022

**Published:** 2020-04-23

**Authors:** Ashley A. Farhat-Sabet, Besher Tolaymat, Antanina Voit, Charles B. Drucker, Rafael Santini-Dominguez, Areck A. Ucuzian, Shahab A. Toursavadkohi, Khanjan H. Nagarsheth

**Affiliations:** ^1^Department of Vascular Surgery, University of Maryland School of Medicine, Baltimore, MD, United States; ^2^Department of Vascular Surgery, Duke University School of Medicine, Durham, NC, United States; ^3^Caribbean Vascular Center, Ponce, PR, United States

**Keywords:** acute limb ischemia, iatrogenic embolization, distal embolization, aspiration thromectomy, penumbra

## Abstract

**Objective:** Acute limb ischemia (ALI) due to thromboembolism is a limb- and life-threatening condition regularly encountered by vascular surgeons. Iatrogenic distal embolization is occasionally seen as a complication of various endovascular procedures. We present a series of four patients who developed ALI due to arterial embolization during cardiovascular procedures that were successfully treated via catheter directed aspiration embolectomy.

**Methods:** Retrospective review of demographics, risk factors, and procedural outcomes was completed for 4 patients who presented with ALI due to distal embolization following cardiovascular procedures. All patients were successfully treated with catheter directed aspiration embolectomy using the Penumbra Indigo System (Penumbra Inc., Alameda, California). All patients had high-quality angiography demonstrating successful embolectomy and end-procedure patency.

**Results:** Three patients presented with Rutherford 2A and one with Rutherford 2B ALI secondary to intraoperative distal embolization. Three patients presented with ALI secondary to distal embolization during peripheral vascular interventions, and one following emergent intra-aortic balloon pump (IABP) placement for myocardial infarction. All emboli were located in the infra-inguinal vasculature. Median post-operative ABIs were 0.94 (*n* = 4). Median length of stay was 2 days. There were no mortalities and no need for adjunctive fasciotomy, amputation, or bypass for limb salvage. All patients improved clinically after intervention, and returned to their reported pre-hospitalization functional status.

**Conclusion:** All procedures achieved technical success with catheter-directed aspiration thrombectomy with or without adjunctive lysis. Catheter-directed aspiration embolectomy with the Penumbra Indigo System for ALI following an iatrogenic embolic event is a safe, less-invasive treatment option. The use of this technology may reduce the need for traditional open thrombectomy or thrombolytic therapy to address ALI.

## Introduction

Acute limb ischemia (ALI) results from an acute decrease in arterial perfusion that threatens limb viability. ALI is frequently of cardioembolic etiology, however it can also result from iatrogenic complications, such as distal embolization of thrombus or atheromatous plaque during peripheral arterial interventions. The acute and long-term morbidity associated with ALI may be devastating, and it has a mortality rate of 9.0% in-hospital, and 15% at 30 days (1). The 5-year survival rate after ALI secondary to acute thrombosis is only 44% (2). Traditional management with a cut-down over the affected vessel and balloon embolectomy has been challenged by effective and less-invasive endovascular approaches (3, 4). Endovascular approaches can include percutaneous transluminal angioplasty, stenting, percutaneous mechanical thrombectomy, pharmacologic catheter-directed thrombolysis, catheter-directed aspiration thrombectomy (CDAT), or a combination of several such techniques (5, 6).

CDAT, traditionally completed by manual syringe aspiration, is an established technique for removal of distal emboli after peripheral vascular intervention (7–10) and new automated devices such as the Indigo System CAT-series catheters (Penumbra Inc., Alameda, CA) have begun to be widely used in recent years as adjuncts to other therapy (11). We present a series of 4 patients who underwent successful CDAT for ALI following apparently iatrogenic distal embolization.

## Methods

### Study Methods

This is a single institution retrospective analysis of a series of cases accrued at an urban tertiary referral center. All data accrual was conducted under the University of Maryland IRB protocol #HP-00085231, which waived consent based on the minimal risks to subjects. The series was developed by chart abstraction for patients identified in administrative data from May through June 2018. The series included 4 patients diagnosed with cardiovascular procedure-associated acute limb ischemia who were treated with CDAT using the Penumbra Indigo system. Patient demographics including comorbidities, and medical treatment were documented (Table 1).

**Table 1:** Patient characteristics.

	**Patient 1**	**Patient 2**	**Patient 3**	**Patient 4**
Age range	66–70	60–65	66–70	80–85
Co-morbidities	HTN, HLD, Smoking	Smoking, CAD	HTN, HLD, smoking	HTN, HLD, CAD, DM, smoking
Race	Black or African American	Black or African American	Black or African American	Black or African American
Pre-operative medical treatment	Statin, beta-blocker	Aspirin, statin, ezetimibe, beta-blocker, diuretic	Clopidogrel, Statin, diuretic	Aspirin, statin, beta-blocker, angiotensin receptor blocker, spironolactone, insulin

*CAD, coronary artery disease; DM, diabetes mellitus; HLD, hyperlipidemia; HTN, hypertension*.

### Procedural Technique

**Figure 1:**
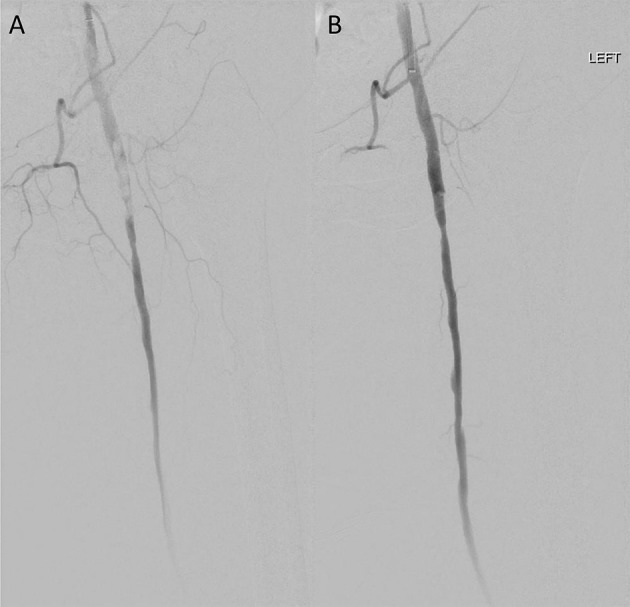
Case 1 intra-operative vascular angiography demonstrating **(A)** iatrogenic distal embolization in the left common femoral artery, and **(B)** resolution of blood flow after percutaneous suction thrombectomy with Penumbra Indigo System (Penumbra Inc., Alameda, California).

**Figure 2:**
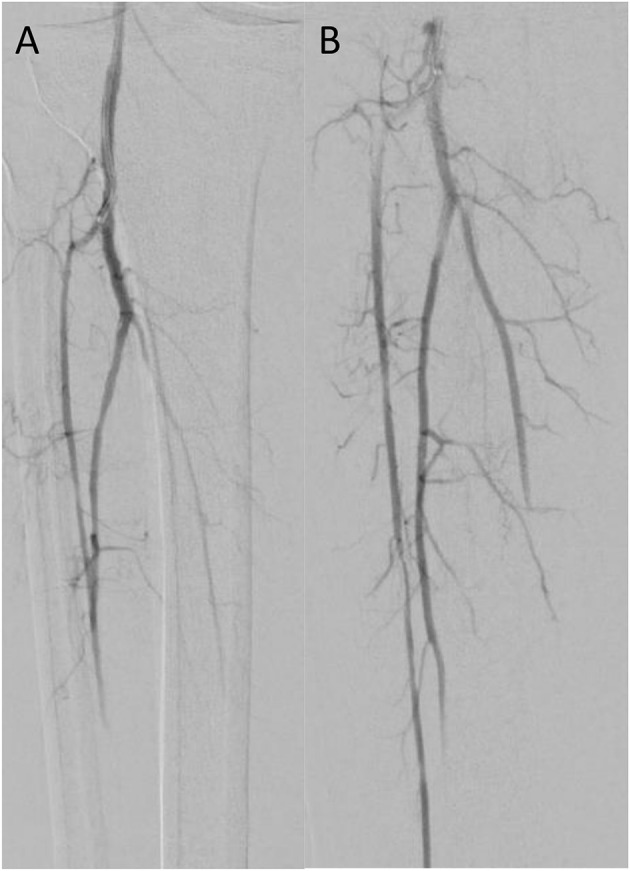
Case 3 intra-operative vascular angiography demonstrating **(A)** iatrogenic distal embolization in the right tibio-peroneal trunk, and **(B)** resolution of blood flow after percutaneous suction thrombectomy with Penumbra Indigo System (Penumbra Inc., Alameda, California).

Computed tomographic or operative angiography was employed preoperatively and intraoperatively to confirm diagnosis. Intraoperative angiography was used to target & deliver therapy and to ensure therapeutic effectiveness. Access was routinely obtained with ultrasound-guided micropuncture of the contralateral retrograde or ipsilateral antegrade femoral access, depending on patient characteristics and ultrasound appearance of access vessels. Initial angiography was used to confirm target anatomy and diagnosis, as well as to guide selection of CDAT catheter. We generally select the 8-French catheter for femoral through popliteal vessels, with selective use of 3, 5, or 6 Fr for mid- and distal-crural arterial targets. Once the site of the lesion is identified, we mark the position on our screen and pass a wire into or across the area of thromboembolic obstruction, which then serves as a guide for positioning the aspiration catheter. The aspiration catheter is placed adjacent to the occlusion. The catheter is connected to the dedicated aspiration system and slowly advanced into the occluding material as the aspirator (“engine”) is activated. Several passes are made before repeat angiography to evaluate the clearance of the occluding material (Figures 1, 2). If there appeared to be considerable residual burden or distal branch disease after 500cc of aspirate was collected, a drip catheter was placed for additional thrombolytic infusion.

Surveillance at follow-up was accomplished by routine use of Ankle-Brachial Indices (ABIs), arterial duplex, and doppler waveform analyses, based on attending preference and initial disease severity (Table 2).

**Table 2:** Peri-operative characteristics and follow-up.

	**Patient 1**	**Patient 2**	**Patient 3**	**Patient 4**
Precipitating event	Right SFA stent placement	Coronary artery catherization, LCX stenting, and IABP placement following a STEMI	Right iliac artery stent placement	Left lower extremity angiogram, balloon angioplasty of occluded SFA and popliteal artery
Rutherford classification	IIB	IIA	IIA	IIA
Location of occlusion	Peroneal artery	Popliteal artery	Tibio-peroneal trunk	Profunda femoris artery, SFA
Additional intervention	Catheter-directed thrombolysis	Left profunda femoris artery repair s/p IABP placement into profunda femoris artery	None	Balloon angioplasty of the Left popliteal artery and left SFA
Follow-up (days)	295	299	130	194
Intra-operative antiplatelet/antithrombotic treatment:	Local TPA, systemic IV heparin, heparin/TPA infusion through lysis catheter	Systemic IV heparin	Systemic IV heparin	Local TPA, systemic IV heparin
Post-operative antiplatelet/antithrombotic treatment:	Heparin Drip	None	Clopidogrel	None

*CABG, coronary artery bypass graft; STEMI, ST elevation myocardial infarction; SFA, superficial femoral artery; TPA, tissue plasminogen activator; IABP, intra-aortic balloon pump; LCX, left circumflex artery*.

## Cases

Four patients were identified with intra-operative ALI secondary to distal embolization during cardiovascular procedures (Table 2). Three patients presented with Rutherford class IIA and one with IIB ALI; three patients reported symptomatic chronic limb ischemia symptoms in the affected limb prior to these procedures. Three patients suffered ALI after peripheral vascular interventions, and one following emergent intra-aortic balloon pump (IABP) placement for myocardial infarction. All lesions were located in the infra-inguinal vasculature. The Penumbra Indigo CDAT achieved reperfusion in all cases. Adjunctive catheter-directed thrombolysis was performed prior to CDAT in one patient for extensive thrombus burden, and CDAT achieved complete reperfusion upon return to OR. Median length of hospital stay from our intervention was 2 days. There were no mortalities and no significant morbidities associated with the ischemic limbs; no subsequent surgical interventions, such as unplanned repeat endovascular intervention, fasciotomy, amputation, or bypass surgery were required for limb salvage. Ankle-brachial indices after surgery were all >0.90 (*n* = 4). All patients returned to their stated pre-hospitalization functional status and underwent follow-up monitoring including lower extremity arterial duplex or Doppler ultrasound without significant restenosis. There were no post-operative complications.

### Case 1

Patient 1 is a smoker with a history of hypertension (HTN), hyperlipidemia (HLD), and right lower extremity claudication, who presented with acute onset of a cool, painful, and numb left lower extremity shortly after right superficial femoral artery (SFA) angiography via left common femoral (CFA) access. Both left dorsalis pedis and posterior tibial arteries were found to be occluded. We accessed the right CFA, and obtained an angiogram that revealed single vessel runoff with left distal peroneal artery occlusion. We advanced a 6 Fr catheter and attempted manual aspiration embolectomy of the left peroneal artery without improvement in subsequent angiogram. CDAT was then completed with a 5 Fr Penumbra catheter, with partial improvement. We advanced our wire to the peroneal artery but could not cannulate it or the anterior tibial artery due to high residual burden. Subsequently, we elected to use catheter-directed thrombolysis with a UniFuse Infusion Catheter (Angiodynamics, Queensbury, New York) to eliminate residual disease in the left peroneal and tibial arteries. She was admitted to the intensive care unit for monitoring during lytic therapy and returned to the operating room the next day. Angiography revealed left peroneal artery occlusion at the takeoff with patent proximal anterior and posterior tibial arteries. We sequentially passed 6 Fr and 3 Fr catheters down to the level of the foot, clearing the residual thrombus from the peroneal, PT, and DP segments via CDAT. We confirmed adequate peroneal runoff (Figure 1). The patient tolerated the procedure well and was started on aspirin 81 mg and a heparin infusion before transitioning to rivaroxaban on postoperative day 3. Post-operative DP pressure was 119 mmHg (ABI 0.92) and left great toe pressure was 59 mmHg. She had no hemodynamically significant stenosis of lower left extremity arteries on follow-up Doppler ultrasound 295 days later and has had no lasting deficits beyond baseline.

### Case 2

Patient 2 is a smoker who underwent left heart catheterization and placement of two drug-eluting stents for ST-elevation myocardial infarction, during which she became hypotensive and hypoxic. She required placement of an IABP for presumed cardiogenic shock. Post-procedure, the patient reported severe left leg pain and was found to have a left DP pressure of 39 mmHg (index of 0.2), and a left toe pressure of 0 mmHg. Her Rutherford IIA ALI was traced to IABP introduction via the PFA, resulting in partial SFA occlusion and distal embolism to the popliteal artery. We obtained ipsilateral antegrade access down the SFA, and CDAT was performed with a 8 Fr catheter, removing a large amount of clot from the below-knee popliteal and peroneal arteries. Completion angiography demonstrated 3-vessel patency to the foot. Post-operative ABIs showed left leg pressure of 134 mmHg (0.86) and a left great toe pressure of 122 mmHg. She has since had no claudication or rest pain and Doppler ultrasound 299 days later revealed no stenosis at either the left profundal or the peroneal segments.

### Case 3

Patient 3 is a smoker with history of HTN and HLD who was transferred to our facility after sustaining an embolism to his right tibioperoneal trunk during elective right iliac artery stent placement at an office-based lab. Occlusion at the tibioperoneal trunk was confirmed by right lower extremity angiogram. We performed CDAT of the right tibioperoneal trunk and branches with a 8 Fr Penumbra catheter with intra-arterial nitroglycerin administration for vasospasm prophylaxis. Completion angiogram demonstrated vessel patency to the level of the foot (Figure 2). Post-operative ABIs showed a right DP pressure of 135 mmHg (index of 1.13) with a right great toe pressure of 74 mmHg. On follow-up 130 days later, he complained of recurrent right lower extremity pain similar to pre-op status, and new onset left lower extremity pain with prolonged ambulation and was found to have moderate plaque in the left middle SFA causing diameter reduction in 50–75% and a posterior wall plaque in the right tibioperoneal trunk without post stenotic turbulence.

### Case 4

Patient 4 is a smoker with a history of HTN, HLD, DM, coronary artery disease s/p myocardial infarction with multiple coronary stents, and significant peripheral arterial disease with multiple bilateral stents, who underwent angiogram of the left lower extremity for severe claudication. This demonstrated an occluded left SFA that was previously stented to the level of the P2 segment of the popliteal artery. We performed balloon angioplasty of the SFA and popliteal, which was complicated by embolization of thrombotic debris into the PFA. She underwent CDAT of the SFA down through the PFA using a 8 Fr catheter. During recovery, the patient complained of acute left heel pain at rest and had an arterial duplex performed which demonstrated low velocities (13 cm/second) in the PT and she was brought back to the angiography suite. We identified a floating thrombus at the femoral bifurcation with focal occlusions in distal branches of the peroneal branch vessels. We administered 10 mg of alteplase, and passed a 8 Fr catheter and completed CDAT with recovery of thrombotic and embolic material from the CFA, SFA, PFA, popliteal, tibioperoneal, and anterior tibial vessels. Completion angiogram demonstrated resolution at all levels. The patient's functional activity level returned to her baseline. Post-operative ABI study had a right DP pressure of 138 mmHg (0.97), and great toe pressure of 132 mm Hg. Upon follow-up 193 days post-operation, she has continued to have improvement of right lower extremity symptoms.

## Discussion

Although a relatively rare complication, reported in between 1 and 5% of lower extremity endovascular interventions, iatrogenic distal embolization (IDE) of atherosclerotic debris or thromboembolism during cardiac and endovascular procedures is of great concern due to potential major adverse sequelae (12, 13). While they may not always incur clinically apparent effects, macro- and micro-embolization occur frequently. Doppler-signal recordings during infrainguinal peripheral arterial interventional procedures demonstrated distal micro-embolization of the ipsilateral tibioperoneal trunks in 100% of cases, most significantly during stent deployment (14). Macroembolizations recorded during peripheral interventions using an embolic protection device have been reported in between 30 and 58% of cases (15, 16). These macroemboli exceeded 1 mm in 58% and 3 mm in 12% of cases during infra-aortic endovascular procedures (16). IDE during a procedure in a high-risk patient can result in significant associated morbidity and mortality. One study estimated that 68% IDEs from lower extremity vascular interventions required an additional treatment, 57% endovascular and 11% open, to address IDE (12). Furthermore, procedures with IDE were less likely to be successful, and patients in this group had significantly longer hospital length of stay (12). Likewise, the incidence of ALI following open cardiac or thoracic surgery is 1.4–1.7% with resultant mortality rates as high as 45% (17). ALI is also a recognized complication of 0.9–21% of intra-aortic balloon pump (IABP) insertions, especially with patients who have risk factors like peripheral arterial disease and diabetes mellitus (18–20).

Features of the lesion as well as patient characteristics can predispose patients undergoing peripheral endovascular interventions to clinically significant IDE (Table 3). One study found that IDE requiring further mechanical or pharmacologic intervention, as compared to non-clinically significant IDE, were associated with significantly longer lesion lengths (130 ± 90 mm vs. 90.05 ± 104.94 mm), more severe angiographic pretreatment lesion stenosis (91.7 ± 14.8% vs. 85.65 ± 14.26%), reduced pretreatment thrombolysis in myocardial infarction (TIMI) flow (1.21 ± 1.34 vs. 2.15 ± 1.1), a higher severity TASC II (Trans-Atlantic Inter-Society Consensus) lesions (C and D compared with A and B), greater angiographic thrombus burden, and the acute onset of symptoms on presentation (21). IDE has also been found to occur more often in patients treated for critical limb ischemia than for claudication (RR 2.06), for increased number of diseased arteries treated, and interventions conducted urgently (RR 1.9) or emergently (3.4) rather than electively (12, 21). Multiple studies have also addressed co-morbidities that result in higher rates of IDE including diabetes (RR 1.51) and history of prior amputation(s) (12, 21).

**Table 3:** Factors that can lead to iatrogenic distal embolization.

**Intravascular procedures**	**Intravascular devices**	**Patient risk factors**
Vascular device fracture Dislodged atheromatous plaque with direct or delayed embolization Especially during stent deployment Longer lesions with increased pre-treatment stenosis/angiographic thrombus burden Increased number of arteries treated TASC C and D lesions Clot formation on intravascular devices	Pacemaker fractures Device migration	Acuity of symptoms in patients undergoing peripheral endovascular interventions Patients undergoing emergency peripheral endovascular interventions compared to elective or emergent Diabetes History of prior amputation

The risk of IDE is also partially determined by the procedure performed: One study investigating patients with SFA or proximal popliteal artery disease found that interventions for in-stent restenosis, chronic total occlusions, and calcified lesions were associated with higher rates of macroembolization (13). Atherectomy and stent deployment dislodge more atherothroemboli than isolated percutaneous transluminal angioplasty (3). SFA balloon angioplasty with concurrent atherectomy and stenting with concurrent atherectomy were associated with a higher risk of IDE compared to balloon angioplasty with stenting (RR 3.20 and 3.15, respectively) (12, 13). It is worth noting that, when studied prospectively, embolic signal was greatest during stent deployment compared to other steps of SFA interventions (angioplasty, atherectomy, stent positioning) (22).

Historically, treatment for IDE has been a cut-down over the affected vessel with open balloon embolectomy. Horvath and colleagues first reported the use of intra-arterial catheter aspiration as a means to treat iatrogenic embolism associated with angioplasty in 1978 (7). Since then, CDAT by manual syringe aspiration for the treatment of IDE associated with endovascular interventions has been multiply reported (8–11). In these studies, CDAT success rates between 87 and 93% have been reported (8–11). More recently, continuous automated aspiration devices have become available that promised increase technical success rates and limit the need for additional or repeat interventions in those with PAD.

One such CDAT device is the Penumbra Indigo System (Penumbra Inc., Alameda, CA), which was previously used for acute ischemic stroke (23), acute pulmonary emboli (24), and in acute renovisceral occlusion (25). The PRISM trial established the efficacy of the Penumbra device for treatment of peripheral arterial thromboembolism, including those with acute peripheral arterial ischemia, and reported complete or near complete revascularization in 87.2% (68/78) patients (26). This included successful revascularization in 79.5% (31/39) patients who solely underwent CDAT, and 92.5% (37/40) who underwent CDAT as salvage or secondary therapy (26).

Two retrospective studies since the PRISM trial have evaluated the use of Penumbra Indigo aspiration catheters as primary interventions in the treatment of peripheral arterial thromboembolism. In one study, with allowed concomitant use of angioplasty or stent placement, they found that near-complete or complete flow (TIMI 2/3) was achieved in 72.7% (24/33) lesions (27). Adjunctive therapies were more often used for above-the-knee lesions, with 46.2% (6/14) of those lesions requiring angioplasty and 38.5% (5/13) requiring stent placement (27). In the other study, they found technical success, defined as a restoration of blood flow with <50% residual thrombus without need for catheter-directed thrombolysis or open surgery, in 52% (15/29) lesions when used as the main treatment compared to 50% (7/14) lesions when used as adjunctive therapy (28). Results of the INDIAN (Indigo System in Acute Lower-Limb Malperfusion) Registry—a large interventional prospective trial of patients with ALI treated with the Penumbra Indio System—should become available soon to provide additional data (29).

Here, we have shared our institutional experience with one such continuous automated CDAT device, the Penumbra Indigo system, which we believe supports the use of such systems in the treatment of IDE. To our knowledge, there is only one published case series of three patients treated for distal embolization complications from limb salvage interventions using the Penumbra System (30). These patients underwent percutaneous transluminal angioplasty with a balloon catheter for obstruction in the tibial or superficial femoral-popliteal arteries with post-angiography imaging revealing distal embolization. Final angiographic imaging showed complete recanalization with restored normal flow. These results are similar to ours in efficacy of recanalization. Accounting for limited sample size thus far, the aforementioned study and this series together support a greater technical success rate when compared to manual syringe aspiration for IDE.

We employed adjunctive thrombolysis in one of these four patients, with good success. Our rationale in similar cases of acute embolism is that patients with significant residual thrombus burden, including side-branch disease, achieve better outcomes with the lysis of the residual thrombus. We attribute this to lysis of small and microvascular thrombus burden, allowing for improved distal tissue bed perfusion following therapy.

The Penumbra CDAT device—compared to the standard balloon embolectomy—may also limit the degree of endothelial damage incurred by the target vessels and associated thrombotic complications. This continuous automated CDAT also allows the surgeon to precisely treat more distal vessels and prevent additional unrecognized IDE while addressing the embolic lesion in a controlled and directed manner. Complications resulting from balloon embolectomy are well-documented, and include arterial injury and rupture, delayed pseudoaneurysm or arteriovenous fistula formation, and diffuse arterial narrowing secondary to intimal proliferation (31–34). These complicates have been attributed to the pressure and shear forces exerted by the embolectomy balloon on the arterial wall (34). There has also been at least one report of subsequent formation of atheroma attributed to the endothelial trauma from balloon embolectomy (35). Although there are few studies investigating the Penumbra device's impact on the vessel wall or subsequent complications, the suction mechanism may ultimately protect vessels from unnecessary direct force to the wall and disruption of the intimal layer. One study investigated whether the Penumbra device might cause less endothelial denudation than other wall-contact thrombectomy devices for neurovascular intervention, but could not establish statistically significant benefit and this was at the expense of increased vessel wall edema of unclear significance (36). These findings require further investigation, but decreased endothelial disruption could also decrease the risk for local recurrent thrombosis and additional study would be required to investigate what significance vessel wall edema may have on long-term outcomes. Embolic protection devices continue to populate the market attempting to reduce this risk and minimize the amount of dislodged material that reaches distal vessels. However, even with these devices, one study showed that the incidence of significant IDE despite the use of an embolic protection device was as high as 12% (15). The Penumbra system not only successfully removes macroemboli at the site of obstruction, but the continuous automatic suction may also prevent further dissemination of microemboli to distal tissue beds.

We demonstrated that CDAT, specifically using the Penumbra Indigo system, can be used as a successful adjunctive technique for management of IDE in patients following cardiac and endovascular interventions. The major limitation in this study is size and a lack of randomized, matched comparator interventions using other systems and methods. A larger prospective study comparing intra-operative data, and more extensive patient outcome points between IDE treated by CDAT with the Penumbra system, CDAT using mechanical aspiration, and conventional open balloon embolectomy would be necessary to draw more concrete conclusions regarding safety and efficacy and to further characterize which lesions might be best suited for CDAT therapy.

## Conclusion

Catheter-directed aspiration thrombectomy with the Penumbra Indigo System for ALI following an iatrogenic embolic event appears to be a safe and effective percutaneous treatment option in our practice, either alone or in combination with other modalities such as thrombolysis or angioplasty in such patients. The use of this technology may offer an alternative to traditional cut-down with balloon embolectomy or thrombolytic therapy.

## Data Availability Statement

The datasets generated for this study are available on request to the corresponding author.

## Ethics Statement

The studies involving human participants were reviewed and approved by University of Maryland IRB protocol #HP-00085231. Written informed consent for participation was not required for this study in accordance with the national legislation and the institutional requirements. Written informed consent was not obtained from the individual(s) for the publication of any potentially identifiable images or data included in this article.

## Author Contributions

AF-S, BT, AV, and CD performed a literature search and contributed to writing the manuscript. AF-S, BT, AV, CD, and RS-D collected data, and performed data interpretation. All authors contributed to study design. ST, AU, and KN contributed to critical revision.

## Conflict of Interest

The authors declare that the research was conducted in the absence of any commercial or financial relationships that could be construed as a potential conflict of interest.

## Abbreviations

ALI: Acute limb ischemia
CDAT: Catheter-directed aspiration thrombectomy
CFA: Common femoral artery
CTA: Computerized tomography angiography
DP: Dorsalis pedis
Fr: French
HLD: Hyperlipidemia
HTN: Hypertension
IABP: Intra-aortic balloon pump
IDE: Iatrogenic distal embolization
PFA: Profunda femoris artery
PT: Posterior tibial
PTA: Percutaneous transluminal angioplasty
SFA: Superficial femoral artery
TIMI: Thrombolysis in myocardial infarction.

## References

[1] Baril DT Kaushik G Rosen AB. Trends in the incidence, treatment, and outcomes of acute lower extremity ischemia in the United States Medicare population. J Vasc Surg. (2014) 60:669–77.e2. 10.1016/j.jvs.2014.03.244 24768362 PMC4492305

[2] Aune S Trippestad A. Operative mortality and long-term survival of patients operated on for acute lower limb ischaemia. Eur J Vasc Endovasc Surg. (1998) 15:143–6. 10.1016/S1078-5884(98)80135-4 9551053

[3] Elliott JP Hageman JH Szilagyi DE Ramakrishnan V Bravo JJ Smith RF. Arterial embolization: problems of source, multiplicity, recurrence, and delayed treatment. Surgery. (1980) 88:833. 7444764

[4] Fogarty TJ Cranley JJ Krause RJ Strasser ES Hafner CD. A method for extraction of arterial emboli and thrombi. Surg Genecol Obstet. (1963) 116:241. 13945714

[5] de Donato G Setacci F Sirignano P Galzerano G Massaroni R Setacci C. The combination of surgical embolectomy and endovascular techniques may improve outcomes of patients with acute lower limb ischemia. J Vasc Surg. (2014) 59:729–36. 10.1016/j.jvs.2013.09.016 24342067

[6] Nowygrod R Egorova N Greco G Anderson P Gelijns A Moskowitz A. Trends, complications, and mortality in peripheral vascular surgery. J Vasc Surg. (2006) 43:205–16. 10.1016/j.jvs.2005.11.002 16476588

[7] Horvath L Illes I Varro J Complications of the transluminal angioplasty excluding the puncture site complications. In: Percutaneous Vascular Recanalization. Berlin; Heidelberg: Springer (1978). p. 126–39. 10.1007/978-3-642-46381-5_19

[8] Turnipseed WD Starck EE McDermott JC Crummy AB Acher CW Jensen SR. Percutaneous aspiration thromboembolectomy (PAT): an alternative to surgical balloon techniques for clot retrieval. J Vasc Surg. (1986) 3:437–41. 10.1067/mva.1986.avs0030437 2936904

[9] Schleder S Diekmann M Manke C Heiss P. Percutaneous aspiration thrombectomy for the treatment of arterial thromboembolic occlusions following percutaneous transluminal angioplasty. Cardiovasc Intervent Radiol. (2015) 38:60–4. 10.1007/s00270-014-0857-6 24599522

[10] Cleveland TJ Cumberland DC Gaines PA. Percutaneous aspiration thromboembolectomy to manage the embolic complications of angioplasty and as an adjunct to thrombolysis. Clin Radiol. (1994) 49:549–52. 10.1016/S0009-9260(05)82935-6 7955868

[11] Oklu R Ghasemi-Rad M Irani Z Brinegar KN Toner E Hirsch JA. Aspiration thrombectomy using the penumbra catheter. J Vasc Int Radiol. (2015) 26:454–5. 10.1016/j.jvir.2014.11.028 25735532

[12] Ochoa Chaar CI Shebl F Sumpio B Dardik A Indes J Sarac T. Distal embolization during lower extremity endovascular interventions. J Vasc Surg. (2017) 66:143–50. 10.1016/j.jvs.2017.01.032 28366300

[13] Shrikhande GV Khan SZ Hussain HG Dayal R McKinsey JF Morrissey N. Lesion types and device characteristics that predict distal embolization during percutaneous lower extremity interventions. J of Vasc Surg. (2011) 53:347–52. 10.1016/j.jvs.2010.09.008 21129906

[14] Kudo T Inoue Y Nakamura H Sugano N Hirokawa M Iwai T. Characteristics of peripheral microembolization during iliac stenting: doppler ultrasound monitoring. Eur J Vasc Endovasc Surg. (2005) 30:311–4. 10.1016/j.ejvs.2005.04.004 15890546

[15] Wilkins LR Ugas M West J Sabri S Angle J Analysis of distal protection debris to identify high risk peripheral interventions. J Vasc Intervent Radiol. (2015) 26:149 10.1016/j.jvir.2014.10.033

[16] Karnabatidis D Katsanos K Kagadis GC Ravazoula P Diamantopoulos A Nikiforidis GC. Distal embolism during percutaneous revascularization of infra-aortic arterial occlusive disease: an underestimated phenomenon. J Endovasc Therapy. (2006) 13:269–80. 10.1583/05-1771.1 16784313

[17] Folkert IW Foley PJ Wang GJ Jackson BM Bavaria JE Desai ND. Impact of acute postoperative limb ischemia after cardiac and thoracic aortic surgery. J Vasc Surg. (2018) 67:1530–6.e2. 10.1016/j.jvs.2017.09.019 29242071

[18] Severi L Vaccaro P Covotta M Landoni G Lembo R Menichetti A. Severe intra-aortic balloon pump complications: a single-center 12-year experience. J Cardiothorac Vasc Anesthesia. (2012) 26:604–7. 10.1053/j.jvca.2012.01.037 22445181

[19] Busch T Sirbu H Zenker D Dalichau H. Vascular complications related to intraaortic balloon counterpulsation: an analysis of ten years' experience. Thorac Cardiovasc Surg. (1997) 45:55–9. 10.1055/s-2007-1013687 9175219

[20] Sirbu H Busch T Aleksic I Friedrich M Dalichau H. Ischaemic complications with intra-aortic balloon counter-pulsation: incidence and management. Cardiovasc Surg. (2000) 8:66–71. 10.1016/S0967-2109(99)00087-3 10661706

[21] Shammas NW Shammas GA Dippel EJ Jerin M Shammas WJ. Predictors of distal embolization in peripheral percutaneous interventions: a report from a large peripheral vascular registry. J Invasive Cardiol. (2009) 21:628–31. 19966364

[22] Lam RC Shah S Faries PL McKinsey JF Kent KC Morrissey NJ. Incidence and clinical significance of distal embolization during percutaneous interventions involving the superficial femoral artery. J Vasc Surg. (2007) 46:1155–9. 10.1016/j.jvs.2007.07.058 18154991

[23] Kulcsar Z Bonvin C Pereira VM Altrichter S Yilmaz H Lövblad KO. Penumbra system: a novel mechanical thrombectomy device for large-vessel occlusions in acute stroke. Am J Neuroradiol. (2010) 31:628–33. 10.3174/ajnr.A1924 20019113 PMC7964234

[24] Al-Hakim R Bhatt A Benenati JF. Continuous aspiration mechanical thrombectomy for the management of submassive pulmonary embolism: a single-center experience. J Vasc Intervent Radiol. (2017) 28:1348–52. 10.1016/j.jvir.2017.06.025 28941516

[25] Bisdas T Stavroulakis K Beropoulis E Schwindt A Stachmann A Austermann M. Initial experience with the 6-F and 8-F Indigo thrombectomy system for acute renovisceral occlusive events. J Endovasc Therap. (2017) 24:604–10. 10.1177/1526602817710492 28548010

[26] Saxon RR Benenati JF Teigen C Adams GL Sewall LE Trialists PR. Utility of a power aspiration–based extraction technique as an initial and secondary approach in the treatment of peripheral arterial thromboembolism: results of the multicenter PRISM trial. J Vasc Intervent Radiol. (2018) 29:92–100. 10.1016/j.jvir.2017.08.019 29128156

[27] Baumann F Sharpe E III Peña C Samuels S Benenati JF. Technical results of vacuum-assisted thrombectomy for arterial clot removal in patients with acute limb ischemia. J Vasc Intervent Radiol. (2016) 27:330–5. 10.1016/j.jvir.2015.11.061 26803572

[28] Lopez R Yamashita TS Neisen M Fleming M Colglazier J Oderich G. Single-center experience with Indigo aspiration thrombectomy for acute lower limb ischemia. In: Annual International Symposium on Endovascular Therapy (ISET). Hollywood, FL (2020). 10.1016/j.jvs.2019.10.079 31918998

[29] de Donato G Pasqui E Giannace G Setacci F Benevento D Palasciano G. The Indigo System in Acute Lower-Limb Malperfusion (INDIAN) registry: protocol. JMIR Res Protoc. (2019) 8:e9972. 10.2196/resprot.9972 30869648 PMC6437606

[30] Gandini R Del Giudice C Merolla S Chegai F Stefanini M Mechanical thrombectomy to treat intra-procedural distal embolization caused during percutaneous revascularization. J Vasc Intervent Radiol. (2015) 26:149 10.1016/j.jvir.2014.10.034

[31] Stoney RJ Ehrenfeld WK Wylie EJ. Arterial rupture after insertion of a Fogarty catheter. Am J Surg. (1968) 115:830–1. 10.1016/0002-9610(68)90527-8 5649838

[32] Rob C Battle S. Arteriovenous fistula following the use of the fogarty balloon catheter: report of a case. Arch Surg. (1971) 102:144–5. 10.1001/archsurg.1971.01350020054015 5543932

[33] Greenwood LH Hallett JW Jr Yrizarry JM Robison JG Brown SB. Diffuse arterial narrowing after thromboembolectomy with the Fogarty balloon catheter. Am J Roentgenol. (1984) 142:141–2. 10.2214/ajr.142.1.141 6606948

[34] Dobrin PB Jorgensen RA. Balloon embolectomy catheters in small arteries. III. Surgical significance of eccentric balloons. Surgery. (1983) 93:402–8. 6829008

[35] Chidi CC DePalma RG. Atherogenic potential of the embolectomy catheter. Surgery. (1978) 83:549–57. 644447

[36] Gory B Bresson D Kessler I Perrin ML Guillaudeau A Durand K. Histopathologic evaluation of arterial wall response to 5 neurovascular mechanical thrombectomy devices in a swine model. AJNR Am J Neuroradiol. (2013) 34:2192–8. 10.3174/ajnr.A3531 23538407 PMC7964832

